# Simultaneous Determination of Fifteen Polyphenols in Fruit Juice Using Ultrahigh-Performance Liquid Chromatography-Tandem Mass Spectrometry Combining Dispersive Liquid-Liquid Microextraction

**DOI:** 10.1155/2022/5486290

**Published:** 2022-03-23

**Authors:** Yuxiu Li, Zengyang He, Youmei Bao, Qingsheng Zhu, Yong Ning, Zhenfeng Tian, Xiaolan Zhu

**Affiliations:** ^1^The USTC-Anhui Tobacco Joint Laboratory of Tobacco Chemistry, Research Center of Tobacco and Health, University of Science and Technology of China, Hefei 230052, China; ^2^The USTC-Anhui Tobacco Joint Laboratory of Tobacco Chemistry, Center of Technology, China Tobacco Anhui Industrial Corporation, Hefei 230088, China; ^3^School of Economics and Management, Anhui Jianzhu University, Hefei 230601, China

## Abstract

Polyphenols are secondary metabolites of plants and used as effective antioxidants in dietary supplements, whose main sources are fruits, vegetables, and grains. To clarify the content and distribution of polyphenols in different fruit species samples accurately, a rapid and sensitive ultrahigh-pressure liquid chromatography-electrospray ionization-tandem mass spectrometry (UPLC-ESI-MS/MS) method combining dispersive liquid-liquid microextraction (DLLME) was developed for quantitative determination of fifteen polyphenol compounds in fruit juice. In this method, the targets were first extracted from 1 g of fruit juice sample using 10 mL of 80% ethanol solution by ultrasonic-assisted extraction (UAE). Then, 1.0 mL of UAE extracted solution, 60 *μ*L of *n*-octanol and 2.0 mL of H_2_O were performed in the following DLLME procedure. A C_18_ reversed-phase column, ZORBAX SB (100 × 4.6 mm, 3.5 *μ*m), was proposed under gradient elution with 0.1% formic acid aqueous solution and methanol mobile phases for the determination of 15 polyphenols, allowing us to obtain polyphenolic profiles in less than 23.0 min. Under the optimum conditions, the enrichment factors ranged from 162 to 194. The results showed that the 15 polyphenols had linear correlation coefficients (*R*^2^) more than 0.99. The limits of detection (LODs) were between 18.3 and 103.5 ng/g, and the average recoveries were between 96.9 and 116.3% with interday relative standard deviations (RSDs) ranging from 4.4 to 8.2% in all cases. The method was successfully applied to the analysis of real fruit juice samples and presented itself as a simple, rapid, practical, and environment-friendly technique.

## 1. Introduction

Polyphenols are a group of phytochemicals and that are used as effective antioxidants in dietary supplements or as remedies in phytopreparations. They comprise more than 8000 substances with highly diverse structures and molecular masses which range from small molecules such as phenolic acids to big molecules consisting of highly polymerized polyphenolic compounds [1]. The most significant plant phenols include phenolic acids (hydroxyderivatives of benzoic and cinnamic acids) and their esters (chlorogenic and caftaric acids), chalcones, coumarins, flavonoids, stilbenes, and lignans. The main sources of these phenolic compounds are fruits and vegetables as well as certain foodstuffs and supplements including tea, wine, olive oil, chocolate, and many others [2–7]. Moreover, polyphenols are important descriptors of fruit quality because of their contribution to taste, color, and nutritional properties [8]. Epidemiological studies have shown that the intake of plant phenols can influence the health of an individual. A great abundance of these compounds in our diet provides important health benefits mainly based on their antioxidant properties and their probable role in the prevention of various diseases such as skin pathologies, various types of cancer, cardiovascular disorders, and other age-related degenerative pathologies [9–13].

In recent years, there have been lots of related studies on phenolic substances and their fingerprints that can be used to control the quality of fruit juices [14–19]. Juices produced from different types of fruits vary in the composition of polyphenolic compounds, for example, grape and red wine are rich in resveratrol while apple, cherry, mango, apricot, and pear are rich in catechins [20]. Moreover, fruit juice is a complex matrix and contains a lot of water-soluble compounds such as sugars and organic acids. When subjected to UPLC analysis directly, these compounds would be unfavorable to the column and give up interference for separation. In addition, fruit juice was derived from different raw materials and subjected to the effects of different processing and storage conditions. Therefore, the content and distribution of polyphenols vary greatly in different fruit juice sources and those polyphenols whose contents are very low need to improve the sensitivity of detection. Thus, the development, validation, and application of new or alternative analytical methods able to characterize the identity of genuine constituents, minimize interferences, and differentiate fruit juice attributes and quality specifications are very relevant.

In the last 20 years, many different approaches and processes have been investigated and used for the enrichment of polyphenols from fruit-based materials such as solvent extraction [21], microwave-assisted extraction [22], biological enzymatic hydrolysis [23], accelerated penetration extraction [24], and ultrasonic-assisted extraction [25]. Among these methods, ultrasound-assisted extraction (UAE) has been successfully used in the extraction of polyphenols from many different matrices, owing to its advantages including lower temperature requirements, less use of solvents, improving the diffusion, solubilisation, and extraction efficiency of the targeted compounds [26]. On the other hand, microextraction techniques have attracted much attention lately due to their simplicity, accuracy, sensitivity, high extraction efficiency, environmental friendliness, and compatibility with a wide range of analytes and analytical instruments [27]. Dispersive liquid-liquid microextraction (DLLME) as an interesting tool for sample pretreatment was first introduced in 2006 by Rezaee et al. [28]. In this technique, a mixture of extractant and dispersant was injected into an aqueous sample to form a water/dispersant/extractant emulsion system which was separated by following centrifugation. Moreover, this technique has extra advantages over other liquid microextraction techniques in terms of rapidity, low cost, simplicity of operation, high recovery, and enrichment factor. So far, as a green and novel microextraction technique, DLLME has been successfully applied to the extraction and preconcentration of various compounds in food [29], medicine [30], environment [31], and other fields. However, there is no report about the enrichment of polyphenols in fruit juice.

The determination of polyphenols can be tackled by various methods mainly including spectrophotometry [32], liquid chromatography (LC) [33], liquid chromatography with mass spectrometry (LC-MS) [34], gas chromatography-mass spectrometry (GC-MS) [35], nuclear magnetic resonance (NMR) [36], capillary electrophoresis (CE) [37], electronic tongues [38]. Among them, liquid chromatography (LC) with UV detection or coupled to mass spectrometry (LC-MS) are the most common techniques described for the determination of polyphenols and the characterization of a great variety of plants and fruit-based products due to their simple sample processing, high sensitivity, and reliable results. Compared with UV detection, MS/MS has become as one of the preferred analytical techniques for quantification purposes providing sufficient sensitivity as well as the capability of unambiguous evidence for phenolic compound identification and quantification at trace levels from a single injection. However, long analysis time is often required in HPLC procedure and ultrahigh-performance liquid chromatography (UPLC) has been used to replace it during the chromatographic separation owing to its several advantages such as better resolution, short run time, and higher sensitivity. Thus, UPLC can be coupled to MS/MS for routine analysis in many complicated matrices [14, 39, 40].

The aim of this study was to develop an UPLC-ESI-MS/MS method for the simultaneous determination of 15 phenolic compounds including gallic acid, neochlorogenic acid, chlorogenic acid, catechin, epicatechin, *p*-hydroxybenzoic acid, caffeic acid, syringic acid, ferulic acid, rutin, coumarin, phlorizin, resveratrol, quercetin, and cinnamic acid in fruit juice. In addition, this article combined the UAE-DLLME method to extract and enrich the polyphenols in the juice so as to reduce the interference of the complex matrix on the target substances. The conditions of UAE extraction, DLLME extraction, and chromatographic separation of UPLC-MS/MS were studied and optimized in detail. Finally, the developed method was validated and applied to the analysis of these polyphenol compounds in real fruit juice samples.

## 2. Experimental

### 2.1. Reagents and Materials

Analytical standards of gallic acid, neochlorogenic acid, chlorogenic acid, catechin, epicatechin, *p*-hydroxybenzoic acid, caffeic acid, syringic acid, ferulic acid, rutin, coumarin, phlorizin, resveratrol, quercetin, and cinnamic acid were purchased from J&K Technology Co. Ltd. (Beijing, China). Deuterated ferulic acid (DFA) was obtained from Sigma-Aldrich (Steinheim, Germany) and used as an internal standard (IS). LC-MS grade water and methanol as well as formic acid (98–100%) were obtained from Tedia Company Inc. (Fairfield, OH, USA). Mobile phases for UPLC were filtered through 0.22 *μ*m membranes. *n*-hexanol, *n*-octanol, *n*-decanol, *n*-dedecanol, acetone, ethanol, and sodium chloride, all of analytical grade, were purchased from Sinopharm Chemical Reagent Co., Ltd. (Shanghai, China).

Fruit juice samples including mulberry, tomato, raisin, blueberry, jackfruit, pear, apple, passion fruit, fig, and cherry juice were obtained from China Tobacco Anhui Industrial Co., Ltd. All juice samples were kept in a refrigerator at 4°C before use.

### 2.2. Instrumentation and Chromatographic Conditions

Chromatographic separation was performed on an ultrahigh-pressure liquid chromatography (UPLC) system (Agilent 1290 Infinity II, Agilent Technology, USA), equipped with a quaternary pump and an autosampler. A ZORBAX SB-C_18_ (4.6 mm × 100 mm, 3.5 *μ*m) reversed-phase column was used for the proposed methods. In addition, there were some small instruments such as electronic analytical balance, ultrasonic instrument, and centrifuge used for this work.

Gradient separation was achieved using solvent A (0.1% formic acid aqueous solution) and solvent B (methanol) at a flow rate of 0.4 mL/min. A gradient program was used within a total time of 30 min: 0.0–4.0 min, 10% B; 4.0–25.0 min, 10–100% B; 25.0–26.0 min, 100-10% B; 26.0–30.0 min was kept at 10% B. The column was kept at 30°C, and the injection volume was 5 *μ*L.

The UPLC system was coupled to an Agilent 6460 Triple quadrupole mass spectrometer, equipped with an ESI probe. The ionization method is electrospray ionization in both positive and negative mode. The temperature of the ionization source was 350°C. The dry gas flow was 10 L/min, the nebulizer pressure was 15 psi, the capillary voltage was 4000 V, the fragmentor was 135 V, the cell accelerator voltage was 7 V, the cycle time was 200 ms, and nitrogen was used as collision gas. The detection was carried out in dynamic-multiple-reaction monitoring (dynamic-MRM) mode with *m/z* parameters shown in Table 1, and the peak area was used for quantification. As can be observed, several polyphenols showed two retention times, such as neochlorogenic acid or resveratrol, and attributed to their structural isomers. The specific time window for each compound (retention time) was set at 1 min.

### 2.3. UAE-DLLME Procedure

Sample treatment was carried out as following: for the UAE step, 1 g of the sample was extracted using 10 mL of 80% ethanol solution (v/v) containing DFA (20 *μ*g/mL) for 20 min in a conical flask assisted by UAE, then kept still for 10 min. For the DLLME, the ethanol extract (1 mL) was removed from the conical flask and rapidly inserted into a 5 mL centrifuge tube containing 2.0 mL of distilled water. After the pH value of the mixture was adjusted to 5.0 with hydrochloric acid (0.1 mol/L), 60 *μ*L of extraction solvent (*n*-hexanol) was injected rapidly into the aqueous solution and a cloudy solution consisting of very fine droplets was formed in the test tube. Then, the mixture was under ultrasonication for 10 min and centrifuged at 4000 rpm for 5 min. Finally, the extraction phase was transferred to a 250 *μ*L spiry insert open vial, and a 5 *μ*L aliquot was injected into the UPLC-MS/MS for further analysis. For evaluation of the efficiency of UAE and DLLME, 1 g of mulberry fruit sample spiked with 50 *μ*L of IS stock solution (400 *μ*g/mL) and mixed standard stock solution (20 *μ*g/g) was extracted and purified under different conditions.

### 2.4. Method Validation

#### 2.4.1. Linearity

To prepare the primary stock solutions (1 mg/mL), 15 polyphenols were accurately weighed and dissolved in methanol. Then, the stock solutions were mixed and serially diluted with ethanol/H_2_O (80 : 20, v/v) to obtain standard working solutions. Among them, *p*-hydroxybenzoic acid, caffeic acid, syringic acid, ferulic acid, coumarin, phlorizin, resveratrol, quercetin, and cinnamic acid were set at 0.5, 1.0, 2.0, 10.0, 20.0, and 50.0 *μ*g/mL within the range of 0.5–50.0 *μ*g/mL, and gallic acid, neochlorogenic acid, chlorogenic acid, catechin, epicatechin, and rutin were set at 1.0, 5.0, 10.0, 20.0, 50.0, and 100.0 *μ*g/mL within the range of 1.0–100.0 *μ*g/mL. The IS stock solution was prepared in methanol and diluted with ethanol/H_2_O (80 : 20, v/v) to obtain a working solution at a concentration of 400 *μ*g/mL. All solutions were stored at 4°C.

The calibration standard samples were prepared by spiking 50 *μ*L of IS stock solution with 1 mL of standard working solution and subjected to the same DLLME procedure as the fruit juice ethanol extract. Based on the plotting of the peak area ratio to the internal standard versus the spiked concentration, the calibration curves were constructed.

#### 2.4.2. Determination of LOD and LOQ

Limit of detection (LOD) and limit of quantification (LOQ) are two important performance features in method validation. LOD and LOQ strictly related to the magnitude of noise in the measurement system could be determined in different ways [41]. In this work, the LODs and LOQs were estimated using a calibration approach and linear regression and obtained with signal-to-noise ratios (S/Ns) of 3 and 10, respectively [42].

#### 2.4.3. Precision and Recovery

The intraday and interday accuracies were evaluated by the determination of the mulberry juice sample in the same day and in six consecutive days with five replicates. The precision was defined as the RSD between the replicate measurements. The recovery experiment was carried out with mulberry juice sample spiked with two levels (5 and 50 *μ*g/g) of the polyphenol standard, and the sample preparation was carried out as in Section 2.3. The extraction recovery was calculated using the ratio between the difference value of content of the analyte in the spiked sample and that of added in the juice sample.

## 3. Results and Discussion

### 3.1. Optimization of the UPLC-MS/MS Analytical Method

To optimize the separation of 15 polyphenols, weak acid condition is beneficial to increase the ionizing efficiency of polyphenols in ion source. Polyphenols especially phenolic acids would stay their free state in an acidic environment which is favorable for ionization efficiency of the compound during subsequent separation. The effect of 4 levels of formic acid solution (0.05%, 0.1%, 0.2%, and 0.3%, v/v) in mobile phase on the separation of polyphenols was investigated. The results (SFigure 1) showed that signal intensity of 15 polyphenols with 0.1% formic acid was obviously higher than the other three conditions. Therefore, 0.1% formic acid solution in mobile phase was selected for the separation of polyphenols.

**Figure 1:**
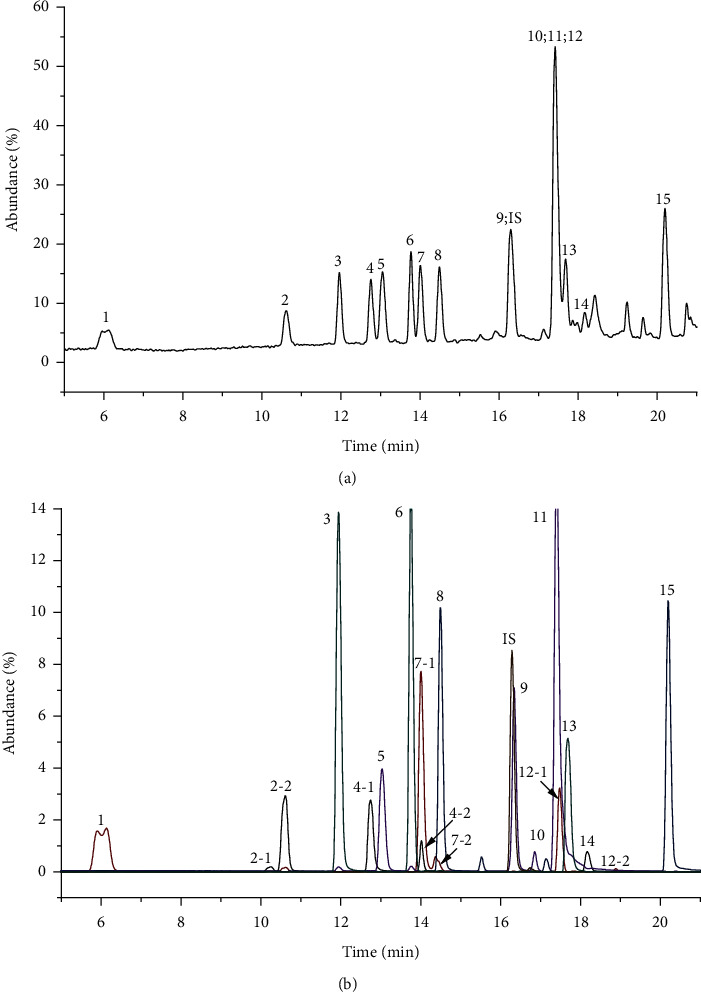
UPLC-MS/MS chromatogram of polyphenolic compounds in full scan mode (a) and MRM scan mode (b). The peak no. was as shown in Table 1, and DFA was used as IS.

Table 1 lists the structure, ion information, and mass spectrometer parameters for the analysed compounds. Among 15 polyphenols, 9 studied polyphenols (gallic acid, neochlorogenic acid, chlorogenic acid, catechin, epicatechin, caffeic acid, coumarin, phlorizin, quercetin, and resveratrol) showed the deprotonated (M-H)^−^ ion while other 6 (*p*-hydroxybenzoic acid, syringic acid, ferulic acid, rutin, and cinnamic acid) showed the deprotonated (M+H)^+^ ion as the base peak of the MS spectra.  Figure 1 shows the UPLC-MS/MS chromatogram of polyphenol standards. It can be seen that the performance of the transfer approach was quite enough and baseline chromatographic separation was near obtained for the analysed polyphenols under the optimal UPLC-MS conditions. However, baseline chromatographic separation is not mandatory because many of these coelutions could be selectively resolved by MS using the appropriate SRM transitions, taking into account that no ion suppression among the studied polyphenols was observed when ESI was used.

### 3.2. Optimization of UAE Solvent

**Figure 2:**
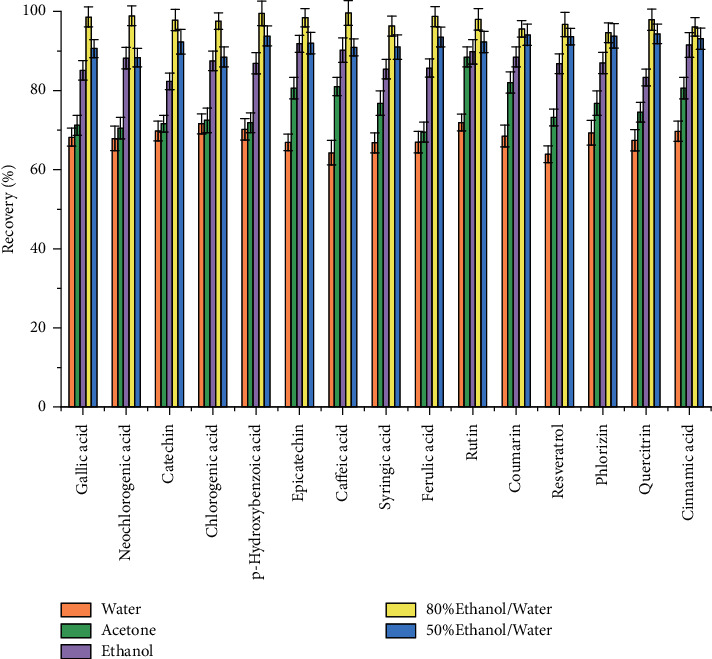
Effect of extraction solvent type on the ER% of polyphenols.

In general, physicochemical properties such as polarity, boiling point, and density (influences penetration into the matrix) are considered when determining the choice of extraction solvent. In our study, water, acetone, ethanol, 80% ethanol/water (v/v), and 50% ethanol/water (v/v) were selected and the extraction efficiencies from fruit juice sample were studied and compared. It was found that the yields of polyphenols extracted by ethanol from fruit juice samples were obviously higher than those by water and acetone (Figure 2). As shown in Table 1, there are many hydroxy groups in the structure of polyphenols with which polar solvents are often used to extract polyphenols for their high compatibility. On the other hand, those nonpolar groups such as phenyl, ester, and hexamethylene groups in the structure of polyphenols made them compatibility with weak polar organic solvents. Acting as an extracting solvent, ethanol molecule has affinities with both polar and nonpolar groups in the structure of polyphenols. Moreover, 80% ethanol/water (v/v) solution showed the highest extraction efficiency and appeared to be the best for the extraction of all the polyphenols.

### 3.3. Optimization of DLLME

To obtain high extraction efficiency, some DLLME important parameters, such as the type and volume of extractant, dispersant, pH value, and salting effect were discussed in detail. Two parameters, enrichment factor (EF) and extraction recovery (ER), were used to evaluate the extraction efficiency under different conditions. The ER% was calculated by the percentage of the mass ratio of polyphenol in the extracted phase to its initial aqueous sample solution. The EF was calculated by the ratio of the concentrations of the analyte in the extracted phase to the initial concentration in the aqueous sample solution.

#### 3.3.1. Selection of Type and Volume of Extractant

**Figure 3:**
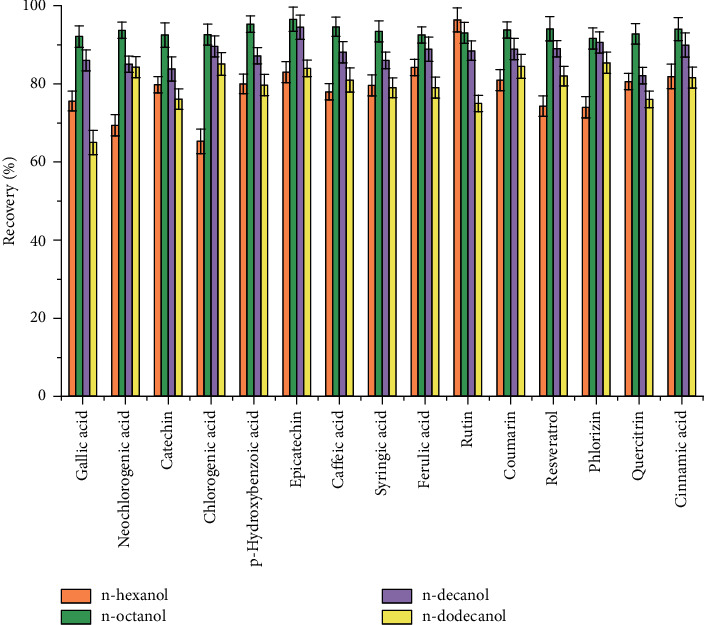
Effect of extraction solvent type on the ER% in DLLME.

The extractant is an important factor affecting the extraction efficiency. In DLLME, the extractant must be insoluble in water and have good solubility for the target to ensure a higher extraction rate. In the experiment, four water-insoluble extractants including *n*-hexanol, *n*-octanol, *n*-decanol, and *n*-dodecanol were selected as extractants. Since the density of the four extractants was at the range of 0.81–0.84, the extract phase was in the upper liquid after centrifugation and transferred by using a micropipette to a 250 *μ*L spiry insert open vial for analysis. The results (Figure 3) showed that all four extractants can extract polyphenols from the aqueous solution in various degrees, and *n-*octanol showed the highest extraction efficiency for the analytes. It is probably due to the lowest solubility in water and most appropriate polarity of *n-*octanol, which has affinities with both polar and nonpolar groups in the structure of polyphenols. Consequently, *n*-octanol was selected as the extraction solvent.

The effect of the extractant volume was examined as follows: different volumes of *n*-octanol (40, 50, 60, and 70 *μ*L) were subjected to the same DLLME procedure. As the results showed, the ER% of polyphenols increased with the volume of *n*-octanol increasing from 40 to 60 *μ*L and remained almost constant when the volume increased from 60 to 70 *μ*L (SFigure 2(a)). However, the EF decreased (SFigure 2(b)) due to the increasing of *n*-octanol volume. In order to obtain the higher EF and ER%, the extractant volume was set to 60 *μ*L in DLLME.

#### 3.3.2. Selection of the Dispersant

In DLLME, dispersant is another important factor affecting the selection of target analytes, which can disperse the extraction solvent as very fine droplets in the aqueous phase. Therefore, the miscibility of dispersant in the aqueous phase and the extract phase is a key index to inspect. In our test, 1 mL of the sample extract (80% ethanol/water solution) contained 0.8 mL of ethanol and was added to 2.0 mL of distilled water (aqueous phase). Because of the favorable compatibility of ethanol with both the aqueous phase and the extract phase (*n*-octanol), 0.8 mL of ethanol had the property of disperser solvent and no extra dispersant was added.

#### 3.3.3. Selection of the pH Value of Aqueous Phase

Phenolic acids and flavonoids are the two main classes of plant polyphenols, and 8 of the 15 analytes (as shown in Table 1) are phenolic acids, which belong to weak acid electrolyte. Therefore, the pH value of the aqueous phase in the extraction system has a specific effect on the ionization status and distribution of polyphenols in two insoluble phases. The effect of different pH values (pH = 2, 5, 6, and 7) of the aqueous phase on the ER% of polyphenols was investigated under the abovementioned optimum conditions. The results (SFigure 3) showed that the ER% of polyphenols increased when the pH value increased from 2 to 5. The weakly acidic environment of the aqueous phase in the extraction system was beneficial to restrain the ionization of polyphenols and maintain their electrical neutrality and therefore increase the partition coefficient of polyphenols in extraction solvent. However, strong acid condition would promote the hydrolysis of some polyphenols, while alkaline condition would lead to acid-base reaction, which both decrease the ER% of polyphenols. Therefore, the optimal pH value of the aqueous phase was 5.

#### 3.3.4. Salting Effect

Salting effect is another important parameter influencing the EF and ER% in DLLME procedure. To determine the effect of salting in the DLLME procedure, different amounts of NaCl (0, 2, 5, and 10%, w/v) were investigated in the test. Salting may decrease the solubility of both polyphenols and *n*-octanol in water, thus increasing the volume of organic phase. The result showed that the volume of the organic phase was 54 *μ*L, 57 *μ*L, 61 *μ*L, and 66 *μ*L with the ratio of NaCl at 0, 2, 5, and 10%, respectively. However, the solubility of polyphenols in *n*-octanol showed no obvious changes and the ER% remained almost constant with the increase of ion concentration. The EF decreased as the volume of the organic phase increased (SFigure 4). To obtain the higher EF, DLLME was carried out without salting.

### 3.4. Method Validation

#### 3.4.1. Calibration Curve Linearity and Sensitivity

As present in Section 2.4, the calibration curves were established using the peak area ratio (*A*_*i*_/*A*_*is*_) and six measured concentration ratios (*C*_*i*_/*C*_*is*_) of each analyte under optimized DLLME conditions. The characteristics of the calibration curves are summarized in Table 2. The correlation coefficients of least-squares regression for 15 polyphenols were bigger than 0.990 at their proper linear dynamic ranges. Because of the outstanding purification and enrichment properties of DLLME technique, the enrichment factors for 15 polyphenols were in the range of 162–194 after the optimized DLLME procedure in this work. As shown in Table 2, LODs and LOQs of the validated method ranged from 18.3 to 103.5 ng/g and 54.9 to 310.6 ng/g, respectively. Compared with other literature reports [43, 44], this method has not only higher sensitivity than the HPLC methods, but also gives the contents and distribution of 15 polyphenols. Therefore, the developed method was far beyond the requirements of the National Food Testing Standard on Foods (GB/T 8313–2018) in China [45] and suitable for analysis of polyphenolic compounds in fruit juice samples.

#### 3.4.2. Recovery and Precision

As presented in Table 3, the recoveries of the 15 polyphenols at two concentrations were from 96.9 to 116.3%, which met the requirement of multi-component detection. The intraday repeatability was within the acceptable range, ranged from 2.23 to 4.52% of RSD, whereas interday repeatability ranged from 4.43 to 8.21% of RSD. Therefore, this method has a high recovery efficiency and sufficient precision for routine analysis and can be applied to the simultaneous detection of 15 polyphenols in real fruit juice samples. The above results also demonstrated that the matrices of fruit juice samples had little effect on the selectivity and the recovery of measurement. That maybe because the application of tandem mass spectrometry would reduce the adverse effects of complex matrices and provide reliable results.

### 3.5. Real Sample Analysis

The proposed UAE-DLLME-UPLC-MS/MS method has been applied to analyze 10 juice samples from China Tobacco Anhui Industrial Co., Ltd. As shown in Table 4, phenolic acids such as *p*-hydroxybenzoic acid, chlorogenic acid, coumarin, and syringic acid are the most widely available polyphenols in fruit juices. Rutin in mulberry juice is the highest polyphenol (420.87 *μ*g/g) and mulberry juice contains the most content (1150.40 *μ*g/g) and species (14 polyphenols) of these juice samples. On the other hand, some sweet fruit juice samples such as pear and blueberry juice have fewer species and lower phenolic acid and flavonoids content. Therefore, the composition and content of phenolic substances of different varieties of fruit juice are significantly different. Research has shown that polyphenolic compounds are the material basis for the flavor, color, and nutritional properties of fruits, which directly affect the taste and quality of fruits and fruit-based processed foods [8]. Because the composition and content of different varieties of fruit juice are the characteristic properties of fruit juice, we can trace the source of fruit juice samples and identify counterfeits from those data, which makes the analysis of polyphenols in fruit juice even more important [15].

## 4. Conclusions

In this work, an UPLC-MS/MS method combining with UAE-DLLME technique was developed and validated for simultaneous quantitation of fifteen polyphenolic compounds in fruit juice. The targets were first extracted by ultrasonic-assisted extraction (UAE), then purified and enriched by DLLME and quantified using UPLC-MS/MS with negative/positive ion electrospray ionization. Under the optimal conditions, the suggested method is simple, fast, inexpensive and environmentally friendly with satisfactory recovery and reproducibility in polyphenol extraction and preconcentration from fruit juice samples. Moreover, the method showed high sensitivity and reliability due to the high enrichment power of DLLME and the anti-interference ability of tandem mass spectrometry, which was very important for those low-content components. Therefore, this method has tremendous potential in analysis of composition and content of polyphenols in food industry.

## Figures and Tables

**Table 1:** Quantitative information and their related mass conditions of polyphenols.

No.	Compounds	Structure	Precursor/product ion (*m/z*)	Collision energy (eV)	Polarity	Retention time (Rt, min)
1	Gallic acid	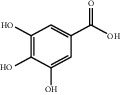	168.9/125.0	10	Negative	6.05
2	Neochlorogenic acid	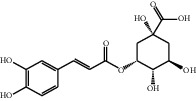	352.8/190.8	15	Negative	10.61^*∗*^, 10.28
3	Catechin	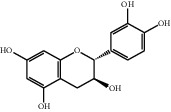	288.8/108.9	20	Negative	11.95
4	Chlorogenic acid	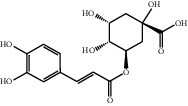	352.8/190.8	10	Negative	12.74^*∗*^, 14.05
5	*p*-Hydroxybenzoic acid	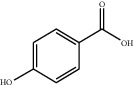	138.9/95.0	10	Positive	13.04
6	Epicatechin	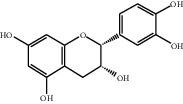	288.8/108.9	20	Negative	13.76
7	Caffeic acid	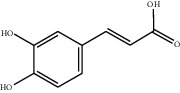	179.0/135.0	15	Negative	14.00^*∗*^, 14.40
8	Syringic acid	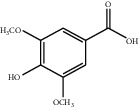	199.0/140.0	10	Positive	14.48
9	Ferulic acid	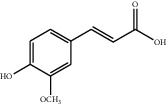	194.9/177.0	5	Positive	16.31
10	Rutin	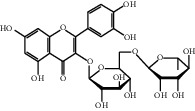	632.9/330.7	30	Positive	17.10
11	Coumarin	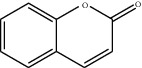	147.0/91.0	25	Positive	17.39
12	Resveratrol	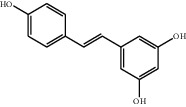	226.9/142.9	25	Negative	17.46^*∗*^, 18.92
13	Phlorizin	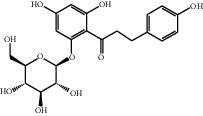	434.8/272.8	5	Negative	17.65
14	Quercitrin	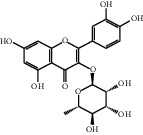	446.8/299.8	20	Negative	18.14
15	Cinnamic acid	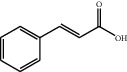	149.0/131.0	5	Positive	20.18

Those analytes with two retention times have isomers, and the peak area labelled was used for quantification.

**Table 2:** Analytical performance data of 15 phenolic compounds by DLLME-UPLC-MS/MS.

Compounds	Linear equation	*R* ^2^	Linear range (*μ*g /mL)	LOD (*μ*g/g)	LOQ (*μ*g/g)	Enrichment factor
Gallic acid	*y* = 0.0939*x* − 0.0194	0.9770	0.91–27.3	0.1035	0.3106	182
Neochlorogenic acid	*y* = 0.0175*x* − 0.0011	0.9908	0.94–47.00	0.0510	0.1531	171
Catechin	*y* = 0.0563*x* − 0.0025	0.9970	1.17–58.50	0.0602	0.1805	176
Chlorogenic acid	*y* = 0.0568*x* − 0.0086	0.9916	1.06–31.80	0.0251	0.0752	172
*p*-hydroxybenzoic acid	*y* = 0.4924*x* − 0.0266	0.9989	1.21–63.25	0.0649	0.1947	169
Epicatechin	*y* = 0.0628*x* − 0.0028	0.9964	0.91–45.50	0.0436	0.1308	174
Caffeic acid	*y* = 1.3728*x* − 0.0169	0.9981	0.90–45.00	0.0384	0.1153	173
Syringic acid	*y* = 0.3124*x* − 0.0384	0.9966	1.35–40.50	0.0724	0.2173	194
Ferulic acid	*y* = 0.9826x + 0.0005	0.9996	1.00–49.75	0.0324	0.0973	169
Rutin	*y* = 0.0222*x* − 0.0013	0.9977	0.92–46.00	0.0379	0.1137	169
Coumarin	*y* = 14.74*x* + 0.5964	0.9948	0.91–22.75	0.0601	0.1803	169
Resveratrol	*y* = 1.9649*x* + 0.1996	0.9854	1.31–32.75	0.0207	0.0621	171
Phlorizin	*y* = 1.8231*x* + 0.0559	0.9965	0.96–24.00	0.0524	0.1572	162
Quercitrin	*y* = 1.9759*x* + 0.1788	0.9726	0.74–18.50	0.0183	0.0549	173
Cinnamic acid	*y* = 1.9043*x* + 0.1355	0.9939	1.06–31.65	0.0446	0.1339	162

**Table 3:** Relative recoveries and RSDs of polyphenols from the spiked mulberry juice sample.

Compounds	Content (*μ*g/g)	RSD (%)		Spiked (*μ*g/g)	Recovery (%)	Average recovery (%)
		Intraday	Interday			
Gallic acid	1.73	3.0	5.3	5	128.0	109.1
				50	90.2	
Neochlorogenic acid	397.72	2.8	4.4	5	104.3	102.4
				50	98.0	
Catechin	10.43	4.3	7.5	5	106.1	105.7
				50	100.6	
Chlorogenic acid	286.97	2.2	6.9	5	109.7	103.4
				50	100.4	
*p*-Hydroxybenzoic acid	4.89	3.8	5.5	5	108.6	101.6
				50	99.5	
Epicatechin	9.06	3.2	4.8	5	103.3	104.1
				50	99.8	
Caffeic acid	9.49	4.3	8.2	5	107.3	103.6
				50	99.9	
Syringic acid	6.04	3.2	4.9	5	109.6	116.3
				50	123.0	
Ferulic acid	0.79	3.5	6.9	5	103.2	101.5
				50	99.8	
Rutin	420.87	2.6	4.5	5	104.3	101.1
				50	98.0	
Coumarin	1.00	3.5	5.1	5	95.7	101.2
				50	108.8	
Resveratrol	—	3.1	5.3	5	93.1	102.8
				50	101.1	
Phlorizin	0.11	3.7	8.1	5	98.0	97.1
				50	107.6	
Quercitrin	0.31	3.6	4.9	5	90.5	103.6
				50	116.7	
Cinnamic acid	0.99	4.5	5.8	5	97.9	96.9
				50	96.0	

**Table 4:** The content of polyphenols analyzed by proposed method in fruit juice samples (*μ*g/g).

Compounds	Mulberry	Tomato	Raisin	Blueberry	Jackfruit	Pear	Apple	Passion fruit	Fig	Cherry
Gallic acid	1.73	1.54	1.55	—^a^	1.59	1.55	1.54	2.44	1.54	1.58
Neochlorogenic acid	397.72	3.93	5.21	8.01	103.76	2.91	16.30	31.51	5.92	117.13
Catechin	10.43	10.41	4.11	—	3.59	—	—	—	—	—
Chlorogenic acid	286.97	20.03	0.58	14.59	121.97	0.48	248.57	70.24	25.98	10.42
*p*-Hydroxybenzoic acid	4.89	0.97	1.15	2.59	1.79	1.07	0.98	1.36	11.06	1.02
Epicatechin	9.06	8.45	3.99	—	—	—	—	—	—	—
Caffeic acid	9.49	3.19	0.97	0.78	1.95	0.76	1.18	1.71	1.33	2.16
Syringic acid	6.04	2.78	1.83	2.08	2.10	1.59	1.52	2.21	2.17	1.58
Ferulic acid	0.79	0.72	0.61	0.64	0.86	0.48	—	1.22	1.49	0.61
Rutin	420.87	49.71	7.98	—	28.41	6.68	—	7.68	6.87	6.68
Coumarin	1.00	0.91	0.91	0.92	0.92	0.90	0.91	0.90	0.95	0.91
Resveratrol	—	—	1.17	—	—	—	—	1.20	—	—
Phlorizin	0.11	—	—	—	—	—	0.06	—	—	0.27
Quercitrin	0.31	0.27	0.62	—	0.12	0.34	0.15	0.26	—	12.38
Cinnamic acid	0.99	0.67	0.66	0.68	1.08	0.66	—	1.71	1.51	0.68
Total	1150.40	103.58	31.34	30.29	268.14	17.42	271.21	122.74	58.82	155.42

^a^Not detected.

## Data Availability

The data used to support the findings of this study are available from the corresponding author upon request. Some figures for the optimization of UPLC-MS/MS condition, UAE conditions, and DLLME conditions are presented in Supplementary Information.

## Supplementary Materials

Supplementary Materials SFigure 1: effect of acid concentration in mobile phase. SFigure 2: effect of extraction solvent volume on the ER% (a) and EF (b) in DLLME. SFigure 3: effect of pH value on the ER% in DLLME. SFigure 4: effect of salt concentration on the EF in DLLME.
