# Supplementary material for: Simultaneous Determination of Fifteen Polyphenols in Fruit Juice Using Ultrahigh-Performance Liquid Chromatography-Tandem Mass Spectrometry Combining Dispersive Liquid-Liquid Microextraction
Source: Int J Anal Chem. 2022 Mar 23;2022:5486290. doi: 10.1155/2022/5486290 (PMC8967586; doi:10.1155/2022/5486290)
Supplement: Supplementary Materials — SFigure 1: effect of acid concentration in mobile phase. SFigure 2: effect of extraction solvent volume on the ER% (a) and EF (b) in DLLME. SFigure 3: effect of pH value on the ER% in DLLME. SFigure 4: effect of salt concentration on the EF in DLLME. [file 5486290.f1.pdf]

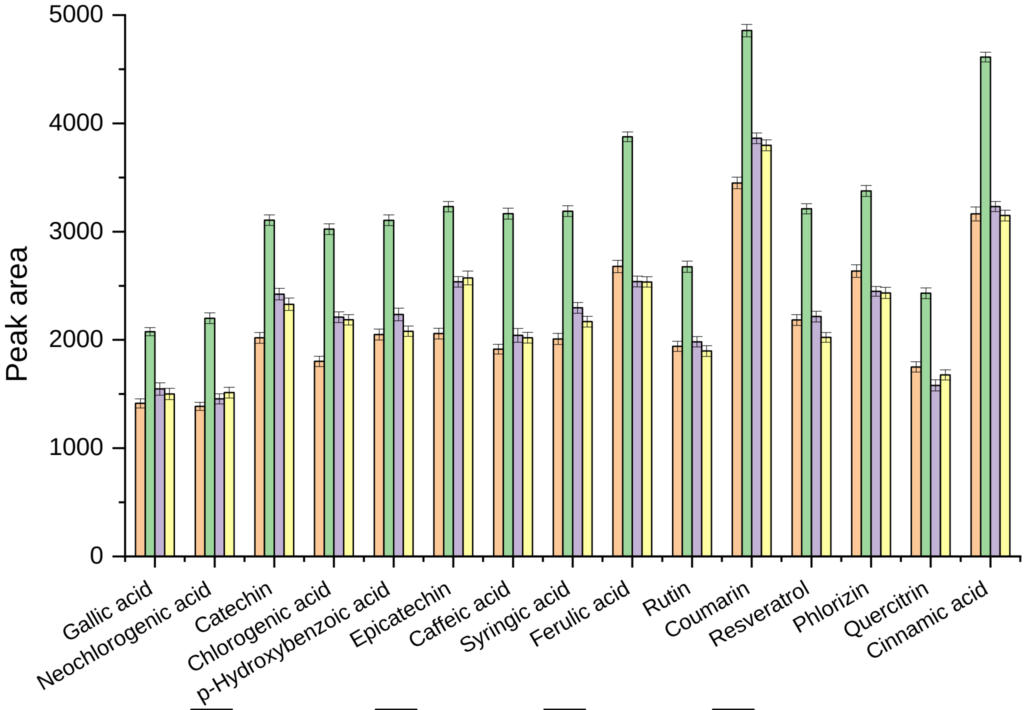

SFIGURE 1. Effect of acid concentration in mobile phase

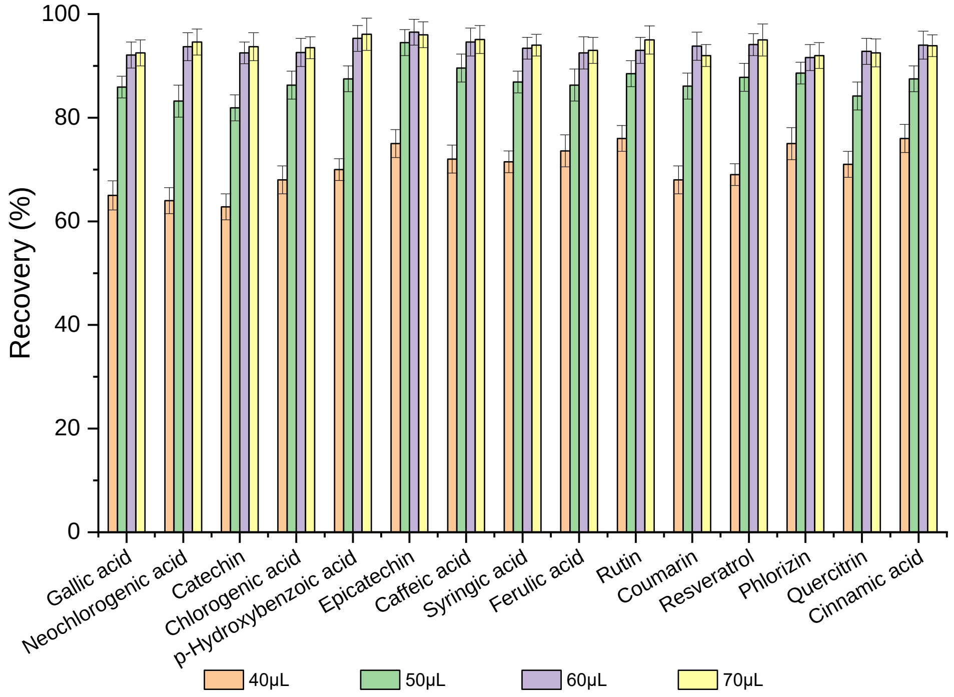

(a)

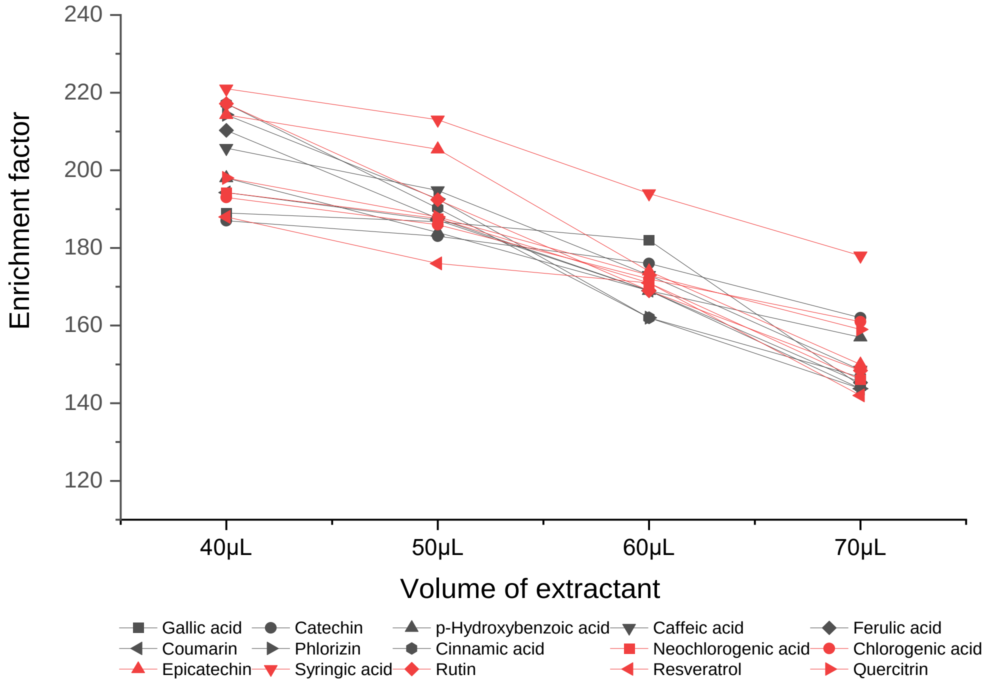

(b)

SFIGURE 2. Effect of extraction solvent volume on the ER% (a) and EF (b) in DLLME (Condition: pH=5; 0%NaCl)

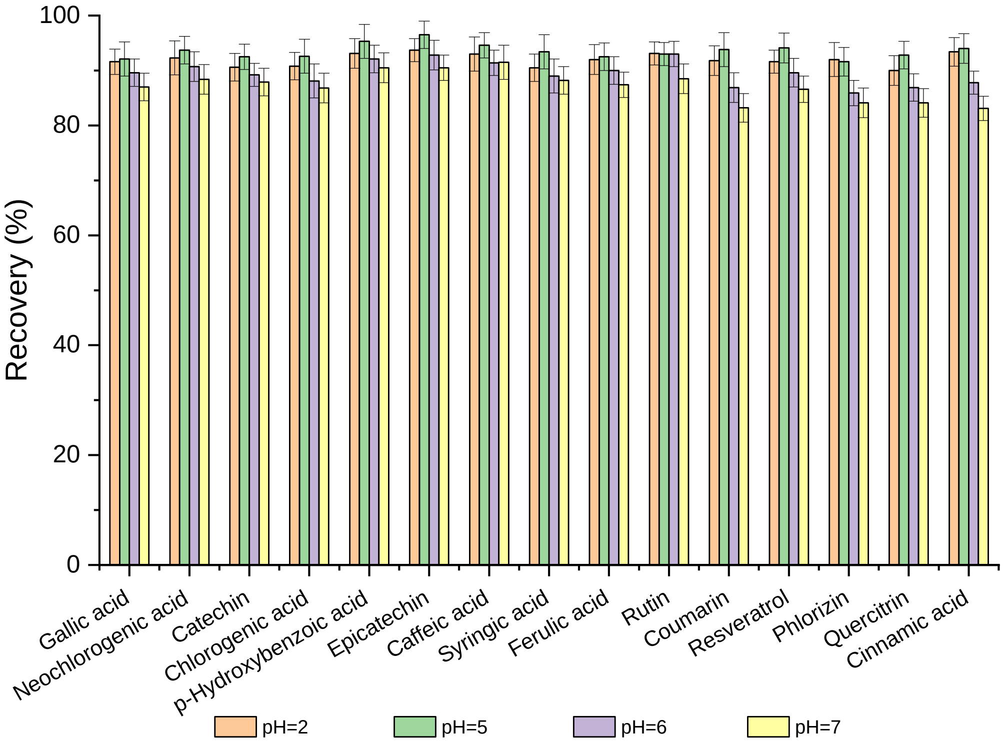

SFIGURE 3. Effect of pH value on the ER% in DLLME (Condition: 60μL extractant; 0%NaCl)

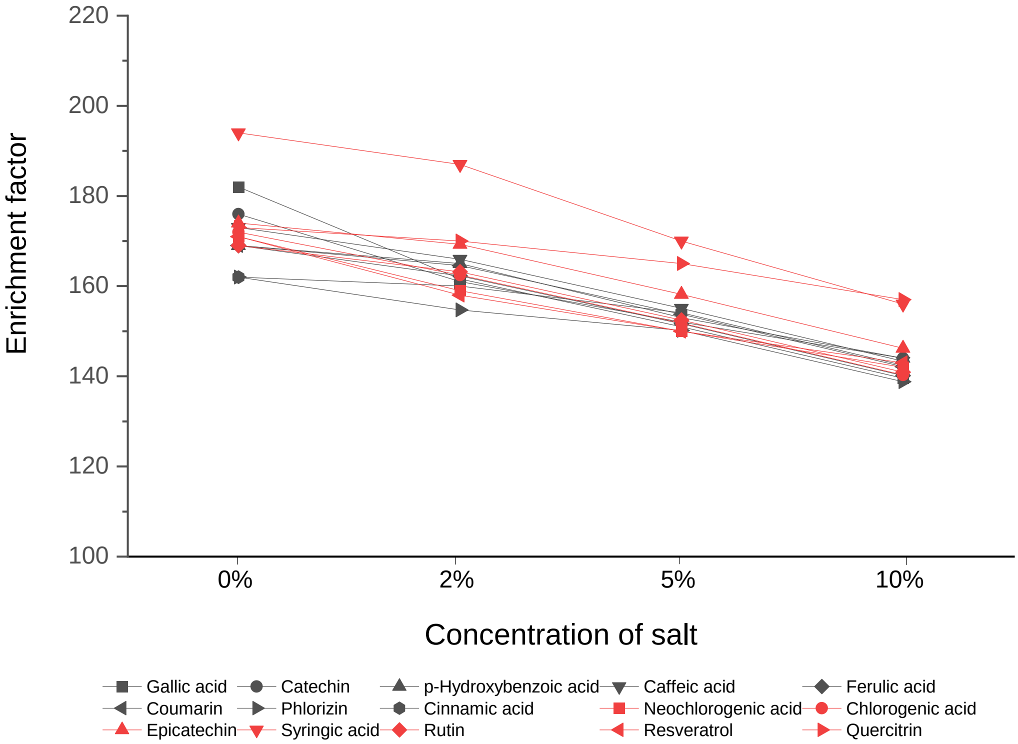

SFIGURE 4. Effect of salt concentration on the EF in DLLME (Condition: pH=5; 60μL extractant)
